# Paclitaxel and Sorafenib: The Effective Combination of Suppressing the Self-Renewal of Cancer Stem Cells

**DOI:** 10.3390/cancers12061360

**Published:** 2020-05-26

**Authors:** Hend M. Nawara, Said M. Afify, Ghmkin Hassan, Maram H. Zahra, Marwa N. Atallah, Hager Mansour, Hagar A. Abu Quora, Md Jahangir Alam, Amira Osman, Hiroki Kakuta, Hiroki Hamada, Akimasa Seno, Masaharu Seno

**Affiliations:** 1 Department of Medical Bioengineering, Graduate School of Natural Science and Technology, Okayama University, Okayama 700-8530, Japan; pnt32hvi@s.okayama-u.ac.jp (H.M.N); pcnp49mj@s.okayama-u.ac.jp (H.M.); pw206tfp@s.okayama-u.ac.jp (M.J.A.); 2 Laboratory of Nano-Biotechnology, Graduate School of Interdisciplinary Science and Engineering in Health Systems, Okayama University, Okayama 700-8530, Japan; saidafify@s.okayama-u.ac.jp (S.M.A.); pthz2c4o@s.okayama-u.ac.jp (G.H.); maram@okayama-u.ac.jp (M.H.Z.); pfgg90pn@s.okayama-u.ac.jp (H.A.A.Q.); mero.osman@med.kfs.edu.eg (A.O.); aseno@wayne.edu (A.S.); 3 Division of Biochemistry, Chemistry Department, Faculty of Science, Menoufia University, Menoufia 32511, Egypt; 4 Department of Microbiology and Biochemistry, Faculty of Pharmacy, Damascus University, Damascus 10769, Syria; 5 Vertebrates Embryology and Comparative Anatomy, Zoology Department, Faculty of Science, Menoufia University, Menoufia 32511, Egypt; marwa.atallah@science.menofia.edu.eg; 6 Cytology, Histology and Histochemistry, Zoology Department, Faculty of Science, Menoufia University, Menoufia 32511, Egypt; 7 Department of Genetic Engineering and Biotechnology, Shahjalal University of Science and Technology, Sylhet 3114, Bangladesh; 8 Department of Histology, Faculty of Medicine, Kafr Elsheikh University, Kafr Elsheikh 33511, Egypt; 9 Division of Pharmaceutical Sciences, Okayama University Graduate School of Medicine, Dentistry and Pharmaceutical Sciences, Okayama 700-8530, Japan; kakuta-h@okayama-u.ac.jp; 10 Department of Life Science, Faculty of Science, Okayama University of Science, Okayama 700-0005, Japan; hamada@dls.ous.ac.jp

**Keywords:** cancer stem cells, combination therapy, paclitaxel, sorafenib

## Abstract

“Combination therapy”, which is a treatment modality combining two or more therapeutic agents, is considered a cornerstone of cancer therapy. The combination of anticancer drugs, of which functions are different from the other, enhances the efficiency compared to the monotherapy because it targets cancer cells in a synergistic or an additive manner. In this study, the combination of paclitaxel and sorafenib in low concentration was evaluated to target cancer stem cells, miPS-BT549cmP and miPS-Huh7cmP cells, developed from mouse induced pluripotent stem cells. The synergistic effect of paclitaxel and sorafenib on cancer stem cells was assessed by the inhibition of proliferation, self-renewal, colony formation, and differentiation. While the IC_50_ values of paclitaxel and sorafenib were approximately ranging between 250 and 300 nM and between 6.5 and 8 µM, respectively, IC_50_ of paclitaxel reduced to 20 and 25 nM, which was not toxic in a single dose, in the presence of 1 µM sorafenib, which was not toxic to the cells. Then, the synergistic effect was further assessed for the potential of self-renewal of cancer stem cells by sphere formation ability. As a result, 1 µM of sorafenib significantly enhanced the effect of paclitaxel to suppress the number of spheres. Simultaneously, paclitaxel ranging in 1 to 4 nM significantly suppressed not only the colony formation but also the tube formation of the cancer stem cells in the presence of 1 µM sorafenib. These results suggest the combination therapy of paclitaxel and sorafenib in low doses should be an attractive approach to target cancer stem cells with fewer side effects.

## 1. Introduction

Cancer is characterized by unlimited growth ability and usually unaffected by growth-preventing signals from other tissues, which results in invasion to local tissues and metastasis to other organs tissues [1,2]. Cancer tissues contain several distinct cellular subpopulations reflecting the heterogeneity arising from a rare population of selected cells [3]. These cells are hypothesized to be cancer stem cells (CSCs) that have tumorgenicity and self-renewal abilities [4,5]. Due to chemo-resistance, chemotherapy usually eliminates the non-CSC population. After chemotherapy, CSCs, which were in a temporary dormant status, are enriched, resulting in tumor relapse and metastasis.

Nowadays the combination therapy is considered a persuasive method to achieve successful chemotherapeutic treatment [6,7]. The combination of multiple anticancer agents sometimes allows the reduction of the drug dose, demonstrating higher response rates, superior safety, and efficacy to monotherapies and avoids the evolution of multi-drug resistance to administered drugs.

The combinations of drugs are applied in recent approaches of precision medicine, focusing on targeting multiple biomarkers found in individual tumors. However, the approaches to target CSCs combining conventional drugs appearing now are ongoing but few are due to the lack of cellular resources [8,9].

Our group recently demonstrated that CSCs could be developed from induced pluripotent stem cells (iPSCs) in the presence of conditioned medium (CM) from cancer cell lines [10,11,12,13,14].

In this study, we evaluated the combination of two different conventional drugs, paclitaxel (PTX) and sorafenib (Sor), employing the CSCs developed from mouse iPSCs in the presence of the CM from human breast cancer cell line BT549 and liver cancer cell line Huh7 [12,14]. PTX is a typical anti-cancer agent extracted from the bark of the Pacific Yew tree, Taxus brevifolia, stabilizing tubulin polymers and preventing disassembly of microtubules. [15]. Sor is a tyrosine-kinase inhibitor broadly used in the treatment of advanced hepatocellular and renal cell carcinoma which suppresses tumor cell proliferation and angiogenesis and promotes tumor cell apoptosis. This effect is due to its activity against the RAF/MEK/ERK signaling pathway. So, to evaluate the effect of the combination using our model is very interesting [16].

## 2. Materials and Methods

### 2.1. Cell Culture

Cancer stem cells, miPS-BT549cmP cells [12] and miPS-Huh7cmP cells [14], were obtained by the conversion of miPSCs (iPS-MEF-Ng-20D-17, Lot No. 012, Riken Cell Bank, Tokyo, Japan), in which the puromycin (puro) resistant gene and green fluorescent protein (GFP) gene were cloned under the control of the Nanog promoter, in the presence of CM from human breast cancer cell line BT549 cells (ATCC HTB-122) and the human liver cancer cell line Huh7 cell line (Riken Cell Bank). All cell lines were with documents confirming their STR profiling.

CSCs were cultured using miPS medium (DMEM media (Wako, Tokyo, Japan) supplemented with 15% fetal bovine serum (FBS), 0.1 mM MEM non-essential amino acids (NEAA) (Gibco, Waltham, MA, USA), 2 mM L-glutamine (Nacalai Tesque, Kyoto, Japan), 50 U/mL penicillin/streptomycin, and 0.1 mM 2-mercaptoethanol (Millipore, MA, USA)) and CM from BT549 cells or Huh7 cell lines, according to the protocol designed by Said et al. [13]. The ratio of the media was 1:1 of miPS medium to CM. CSCs was maintained on 1% gelatin-coated 60 mm-dishes.

### 2.2. Anticancer Drug and Inhibitor

PTX was provided by (Professor Hiroki Hamada) and commercially available Sor (LC laboratories, MA, USA) was purchased from Bayer Pharmaceuticals (Bayer AG, Leverkusen, Germany). Both PTX and Sor were dissolved in dimethyl sulfoxide (DMSO) at the stock of a concentration of 1 mM and stored at −20 °C. Another stock for work diluted in 1× PBS contained DMSO at a concentration of less than 0.1% to prevent background effects on cell growth.

### 2.3. Cell Proliferation and Viability Assay

To measure the viability of cells, cells were seeded in 96-well plates (5000 cells/well) and plates incubated overnight at 37 °C in the presence of 5% CO_2_. Then, the cells were incubated with a series of varying concentrations of PTX, Sor, and combination of them. After 72 h incubation with drug, cell viability was assayed colorimetrically using thiazolyl blue tetrazolium blue (MTT, Sigma-Aldrich, St. Louis, MO, USA). MTT solution was added at a final concentration of 0.5 mg/mL in each well and the plate was incubated for 4 h. Formed formazan crystals were dissolved with 10% (*w*/*v*) SDS in 0.02 N HCl and incubated overnight. Finally, the absorbance of each well was measured at 570 nm [17] using an MTP-800 Lab microplate reader (Corona Electric, Ibaraki, Japan). The experiment was performed in triplicate. Cell viability was calculated relative to the untreated cells. IC_50_ values were estimated from the survival curve.

### 2.4. Sphere Formation

The capacity of self-renewal was estimated by sphere-forming potential in non-adhesive conditions. Both cells were maintained on 96-well low attachment plates (EZ Bind Shut TMSP, Tokyo, Japan) (200 cells/well) in FBS-free DMEM supplemented with insulin-transferrin-selenium-X (ITS-X;1/100 *v/v*) (Life Technologies, Carlsbad, CA, USA), 0.1 mM NEAA, 2 mM L-glutamine 50 U/mL penicillin/streptomycin (Wako), and 0.1 mM 2-mercaptoethanol (Sigma-Aldrich). After 7 days, the number of wells with at least one sphere with diameter ≥50 um was counted. Images were acquired using an IX81 inverted microscope (Olympus, Tokyo, Japan) equipped with a light fluorescence device (Olympus).

### 2.5. Clonogenic Assay

Only a fraction of seeded cells retains the capacity to produce colonies. Before treatment, cells were seeded on gelatin-coated 60-mm dishes (400 cells/dish) to form colonies. After 24 h of incubation, seeding cells were treated with different concentrations of both compounds and their combination. Cells were incubated after treatment for 7 days. Colonies appeared and then were fixed using methanol 75%, stained with Giemsa stain, then colonies with diameter ≥50 um were counted using ImageJ-NIH software.

### 2.6. In Vitro Tube Formation Assay

For in vitro tube formation assay, 12-well plates were coated with growth factor reduced Matrigel (Corning Inc., Corning, NY, USA) and 5 × 10^5^ cells were seeded in 50 μL of endothelial cell growth medium 2 (PromoCell, Heidelberg, Germany) for 24 h with growth supplements: human epidermal growth factor (hEGF; 5 ng/mL), vascular endothelial growth factor (VEGF; 0.5 ng/mL), R3-insulin-like growth factor-1 (R3-IGF-1; 20 ng/mL), ascorbic acid (1 μg/mL), hydrocortisone (0.2 μg/mL), human basic fibroblast growth factor (hbFGF; 10 ng/mL), heparin (22.5 μg/mL), FBS (0.02 mL/mL), gentamicin/amphotericin-B (GA). The drugs were added at same time of seeding and incubated for 24 h. Pictures were taken by Olympus IX81 microscope (Olympus).

### 2.7. Apoptosis Analysis

Level of apoptosis was estimated by flow cytometry using APC Annexin V apoptosis detection kit with propidium iodide (PI) (BioLegend, San Diego, CA, USA) as per the manufacturer’s protocol. Briefly, cells were seeded in 6-cm dishes and incubated overnight. When the cells became confluent around 90% approximately, cells were treated with drugs. After 24 h of drugs treatment, total cells were harvested, washed, stained with APC-Annexin V and PI and analyzed by BD AccuriTM C6 plus flow cytometer (Becton & Dickinson, Franklin Lakes, NJ, USA). Data of each group were analyzed using FlowJo^®^ software (FlowJo, LLC, Ashland, OR, USA).

### 2.8. Flow Cytometry

To detect GFP expression, flow cytometry analysis was conducted on both cultured cells. The cells were seeded and incubated overnight. When the cells became confluent around 90% approximately, cells were treated with drugs. After 24 h of drugs treatment, around (5 × 10^5^) cells were suspended in PBS then GFP expression was estimated using BD AccuriTM C6 Plus. Data were analyzed with Flowjo software (Treestar Inc., San Carlos, CA, USA).

### 2.9. Chorioallantoic Membrane Assay

Angiogenic properties of miPS-Huh7cmP cells and miPS-BT549cmP cells were tested in vivo with a chorioallantoic membrane assay (CAM). Fertilized chicken eggs (Gallus gallus) were incubated in a humidified atmosphere at 37 °C for three days. At day 4 of embryonic age, albumin was removed to detach the developing CAM from the eggshell. At day 8, eggs with exposed CAM were incubated with plastic discs containing 9 × 10^5^ cells pretreated or untreated dissolved in growth factor reduced Matrigel™ droplets (Corning Inc., Corning, NY, USA). Plastic discs containing PBS droplets were used as a negative control. Eggs were incubated for four consecutive days and at day 13, the test was stopped. Images were taken with a Sony HDR-XR350VE Handycam camera (Sony corporation, Tokyo, Japan).

### 2.10. Statistical Analysis

Data from three independent experiments and mean values were presented as mean ± SD at least three-time determinations and analyzed by Student’s *t*-test, as well as one-way analysis of variance (ANOVA). A *p*-value of less than 0.05 was statistically significant.

## 3. Results

### 3.1. Synergistic Effect of PTX and Sor on Proliferation and Self-Renewal

**Figure 1:**
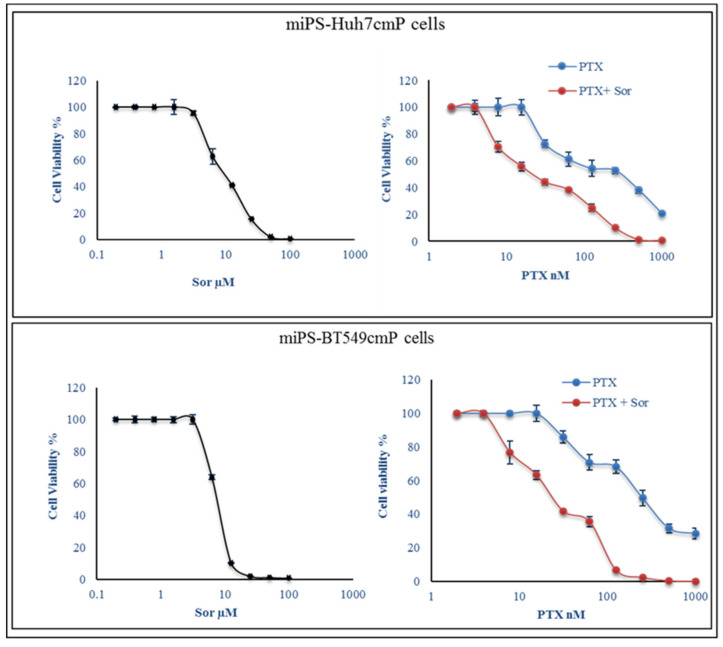
Combined treatment reduced cell viability than monotherapies. The effect of paclitaxel (PTX) and sorafenib (Sor) on cell viability was measured by MTT assay as described in Materials and Methods. Combined treatment inhibited viability in both cell lines in a dose and time-dependent manner. Experiments were performed in triplicate and data are presented as the mean ± SD of three separate experiments.

The IC_50_ values of PTX were 297.9 and 247.5 nM on miPS-Huh7cmP and miPS-BT549cmP cells, respectively, whereas those of Sor were 6.5 and 8.0 µM on miPS-Huh7cmP and miPS-BT549cmP cells, respectively (Figure 1). Although Sor did not show toxic effects up to 5 µM on both cells, the IC_50_ values of PTX on miPS-Huh7cmP and miPS-BT549cmP cells were drastically decreased to 23.6 and 25 nM in the presence of Sor.

**Figure 2:**
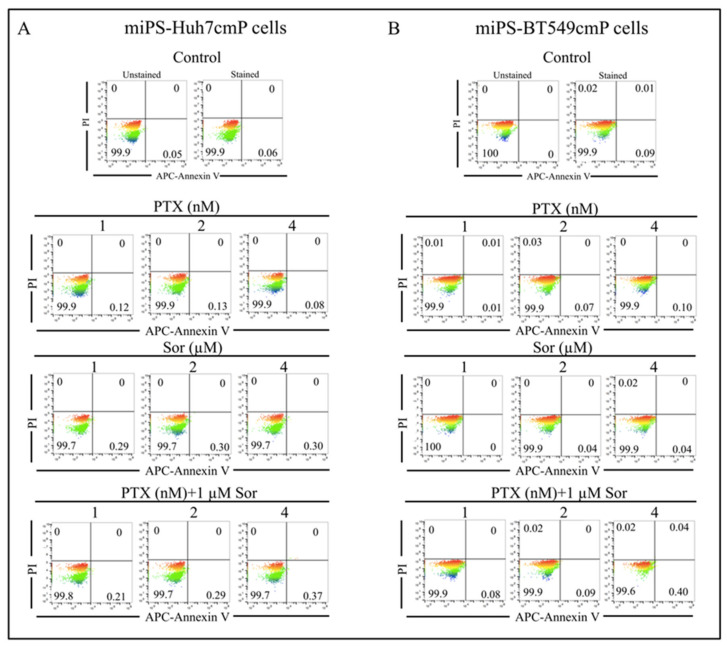
Annexin V-APC/PI double staining flowcytometric analysis of apoptosis in miPS-Huh7cmP (**A**) and miPS-BT549cmP cells (**B**) with/without treatment shows no induction of cell death at concentration 1, 2, and 4 nM of PTX, at concentration 1, 2, and 4 μM for Sor and at 1 μM of Sor with 1, 2, or 4 nM PTX for the combination. Each result is shown as a representative of at least three independent experiments.

In order to evaluate the synergistic effect of the PTX and Sor combination, we used low concentrations of both drugs. For PTX, concentrations were, 1, 2, and 4 nM and 1, 2, and 4 μM for Sor, while the concentrations for the combination were 1 μM Sor with 1, 2, or 4 nM PTX. To confirm that all used concentrations did not induce cell death, we assessed the effects of PTX, Sor, and the combination on the apoptotic status of miPS-Huh7cmP cells and miPS-BT549cmP cells with/without treatment. As the results, the concentrations used in this study and the combination did not induce cell death (Figure 2).

**Figure 3:**
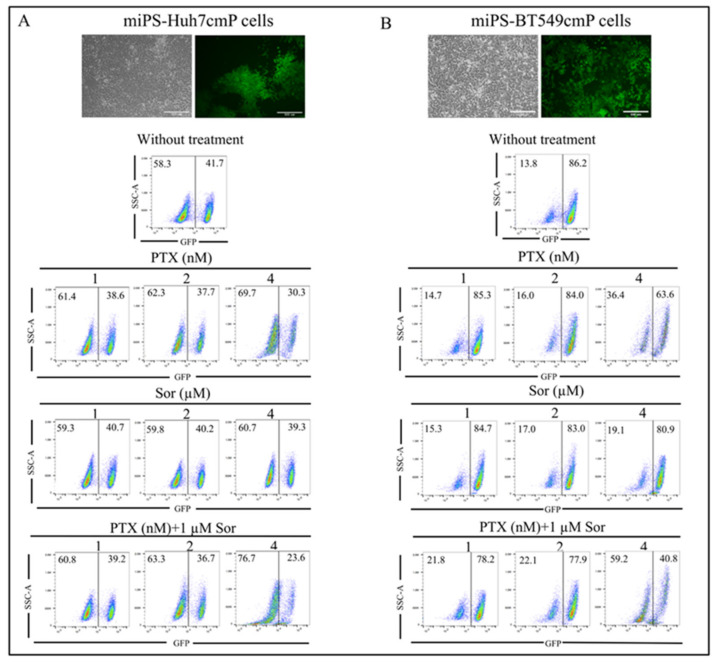
Combined treatment reduced the green fluorescent protein GFP more than monotherapies. Flow cytometry analysis of GFP expressing cells for miPS-Huh7cmP (**A**) and miPS-BT549cmP cells (**B**). Each result is shown as a representative of at least three independent experiments (*n* = 3).

In adhesive culture condition, both cells exhibited two different sub-populations; one was cells expressing GFP which indicates the presence of CSCs as the original miPSCs contained the (puro) resistant gene and (GFP) gene which were cloned under the control of the Nanog promoter, while the second was fibroblast-like cells without expressing GFP. The synergistic effect of PTX and Sor was assessed on the adherent culture and the ratio of GFP-positive and -negative cells was estimated by flow cytometry. As a result, combined treatment reduced the GFP expressing cells percentage from 41.7% in the control to 23.6% in the case of the combination between 1 μM Sor and 4 nM PTX, while in the case of the treatment with 4 nM PTX alone, the percentage of GFP expressing cells was 30.3% for miPS-Huh7cmP cells. The combination between 1 μM Sor and 4 nM PTX exhibited the same manner in the case of miPS-BT549cmP cells in reducing the number of GFP expressing cells, the GFP percentage reduced from 86.8% in the control to 40.8%, while in the case of the treatment with 4 nM PTX alone, the percentage of GFP expressing cells was 63.6% (Figure 3).

**Figure 4:**
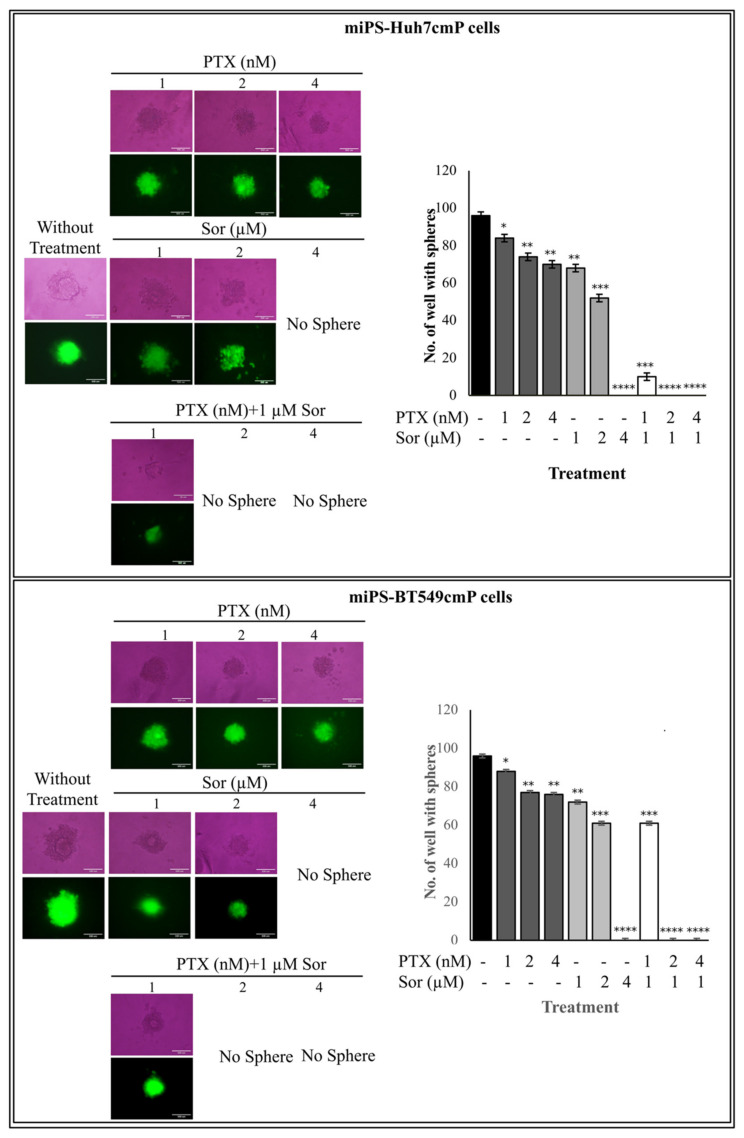
Sphere formation assay from mips-Huh7cmP and mip-BT549cmP cells. Sphere formation potential assessed after cultivation in serum-free cell culture medium. After 7 days incubation, the number of wells with at least one sphere with diameter ≥50 um was counted. The asterisks represent the mean expression levels; * *p* < 0.05; ** *p* < 0.001; *** *p* < 0.0001.

The synergistic effect of PTX and Sor was further assessed on the self-renewal property of both cells as CSC models (Figure 4). The number of spheres was down-regulated to 85–70% by PTX in the range of 1, 2, and 4 nM in a dose-dependent manner, in which concentration, PTX did not show IC_50_ in both cells. Since Sor did not affect at a concentration less than 1 µM, the effect of Sor was assessed from 1 µM. Sor started to suppress the number of spheres at 1 µM and the cells could not form a sphere at 4 µM. To make the doses less to avoid unexpected side effects, we chose Sor at 1 µM, which was found effective in the IC_50_ values as described above, for the combination with PTX to assess the synergistic effect in this study. Again, Sor at 1 µM exhibited the drastic enhancement of PTX at 2 and 4 nM in the regulation of the sphere numbers down to zero, indicating the self-renewal potential of CSCs was completely suppressed. In addition, to confirm the efficiency of this combination more, all doses used in this study were applied to the hepatocellular carcinoma cell line (Huh7 cells). As a result, the number of spheres formed with huh7 cells was reduced to a range of 80–60% by PTX in the range of 1, 2, while in the case of and 4 nM PTX, no sphere formed. In the case of 1 µM Sor, there was not so much difference when compared to 1 nM PTX, while in the case of 2 µM or 4 µM Sor, there was no sphere at all. Then, combination between 1 µM of Sor and 1 nM PTX showed complete inhibition for the sphere formation of Huh7 cells (Figure S1).

### 3.2. Synergistic Effect of PTX and Sor on Colony Formation

**Figure 5:**
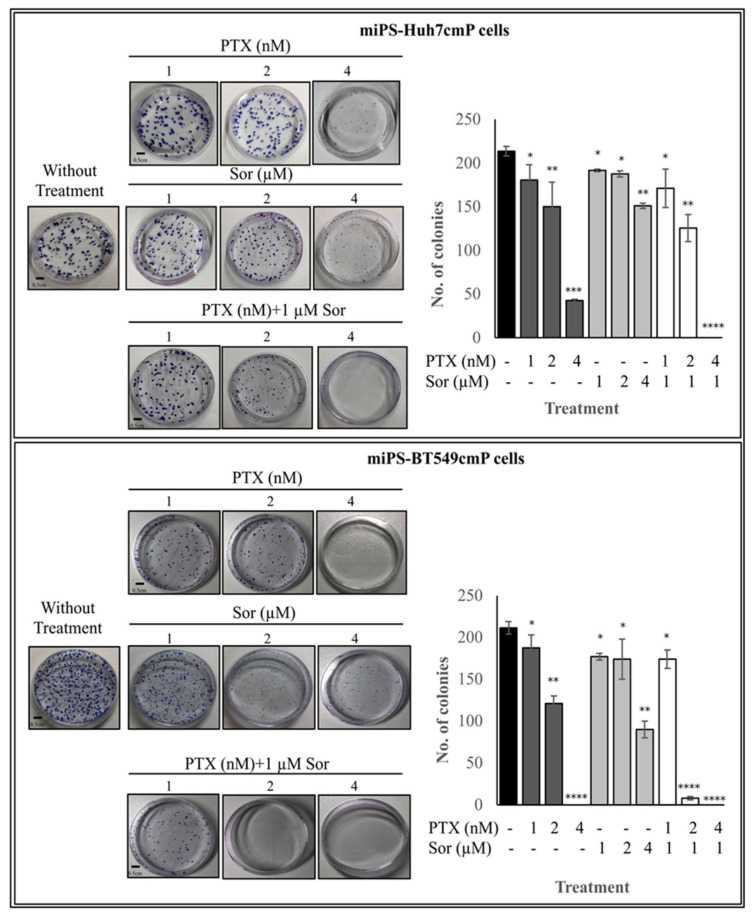
Co-treatment of PTX and Sor affect colony formation potential better than monotherapy. The effect was measured by clonogenic assay using Giemsa stain and colonies with a diameter ≥50 μm were counted. Combined treatment inhibited viability in both cell lines in a dose- and time-dependent manner. All experiments were performed in triplicate and data are presented as the mean ± SD of three separate experiments (* *p* < 0.05; ** *p* < 0.001; *** *p* < 0.0001, **** *p* < 0.00001 vs. the control group).

The synergistic effects of PTX and Sor were further confirmed for colony-forming ability by clonogenic assay (Figure 5). While untreated cells showed more than 200 colonies, PTX at 1 and 2 nM showed a decrease from 90% to 60 % in colony numbers and reached to 0% for miPS-BT549cmP cells and to 40% for miPS-Huh7cmP cells at 4 nM. Simultaneously, Sor decreased the number of colonies down from 90% to 45 % in the range of 1 to 4 µM when compared with untreated cells. Cells treated with PTX together with 1 µM of Sor exhibited a significant reduction in colony numbers. These data illustrate that the low concentration of PTX could suppress colony forming potential more effectively when combined with 1 µM of Sor than PTX could when used alone.

### 3.3. Synergistic Effect of PTX and Sor on Angiogenesis

**Figure 6:**
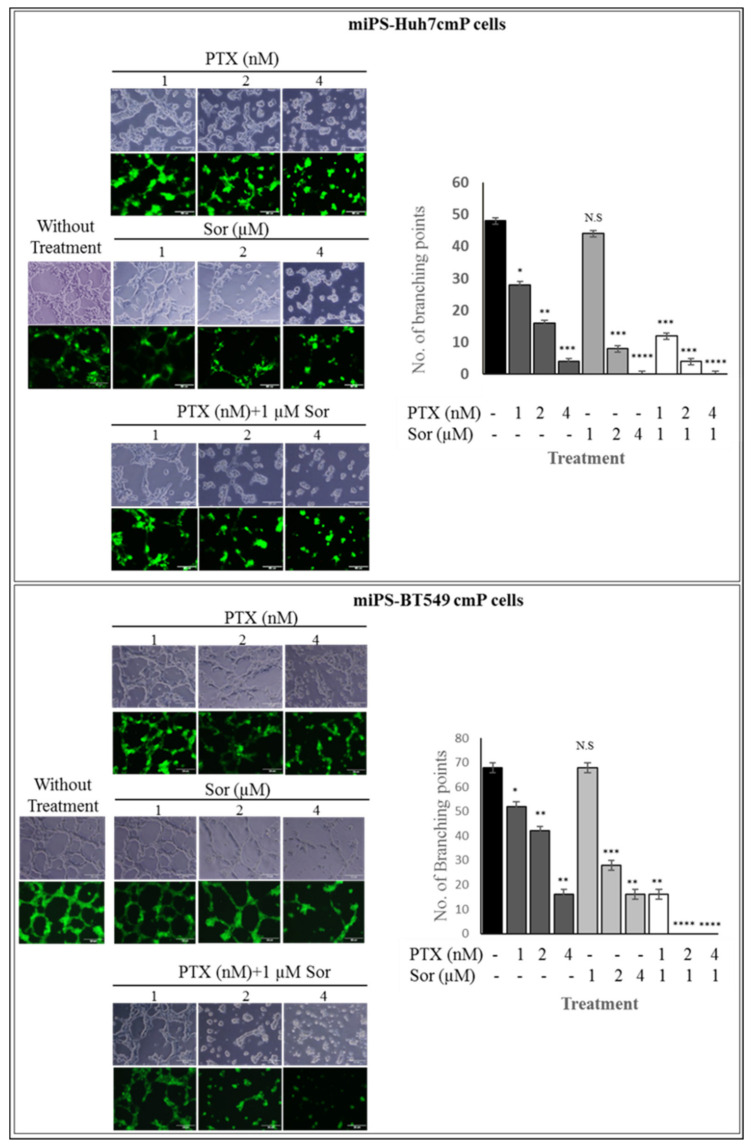
Microscopic images showing tube formation and branching points in each of the three treatment groups: PTX, Sor, and combination between PTX and 1 µM Sor treated group. Branching points was decreased when PTX combined with 1 µM Sor comparing with monotherapy * *p* < 0.05; ** *p* < 0.001; *** *p* < 0.0001, **** *p* < 0.00001).

The effect of the combination of PTX and Sor was assessed for the tube formation (Figure 6). PTX in 1 to 4 nM displayed a potent anti-angiogenic activity. Sor in 1 µM did not affect the number of vessels branching points, while in 2 and 4 µM significantly decreased the number. Interestingly, 1 µM of Sor enhanced the effect of PTX in the CSCs, especially miPS-BT549cmP cells.

**Figure 7:**
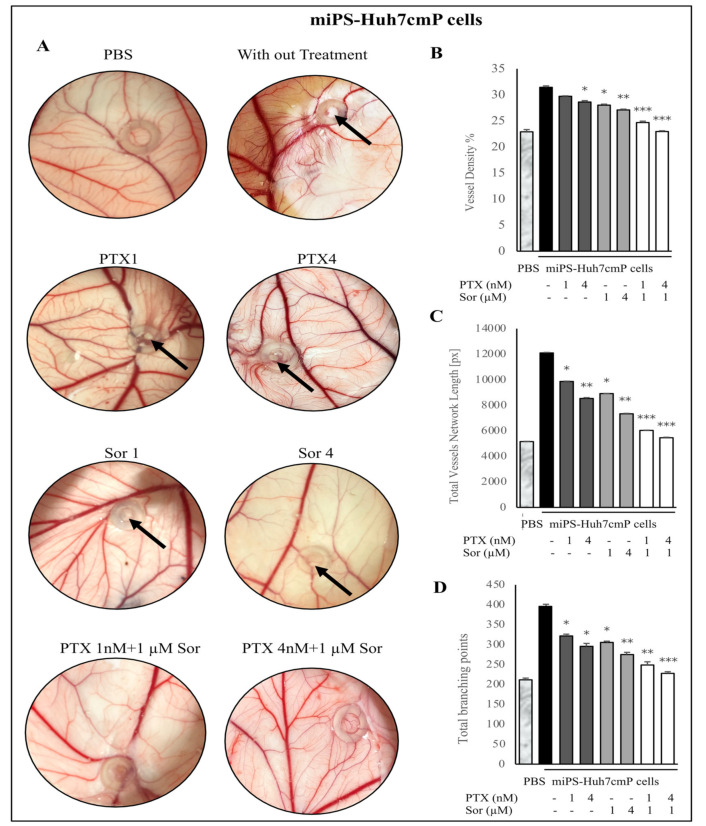
In vivo CAM assay. (**A**) Macroscopic observations of the antiangiogenic effect of combined treatment of PTX and Sor on miPS-Huh7cmP cells. Numerous allantoic vessels converge radially around non-treated controls. Following combined treatment with PTX and Sor, evident reduction in neovessel formation compared with single treatment groups. In addition, inhibition of tumor formation was found in combined groups compared with single treatment groups (black arrow). (**B**–**D**) Analysis of photographs from (**A**) of the vessel density (**B**), total vessel length (**C**), and number of branching points of the vessels (**D**) of the CAM illustrating the anti-angiogenic effect of single or combined treatment of PTX and Sor of miPS-Huh7cmP cells.

**Figure 8:**
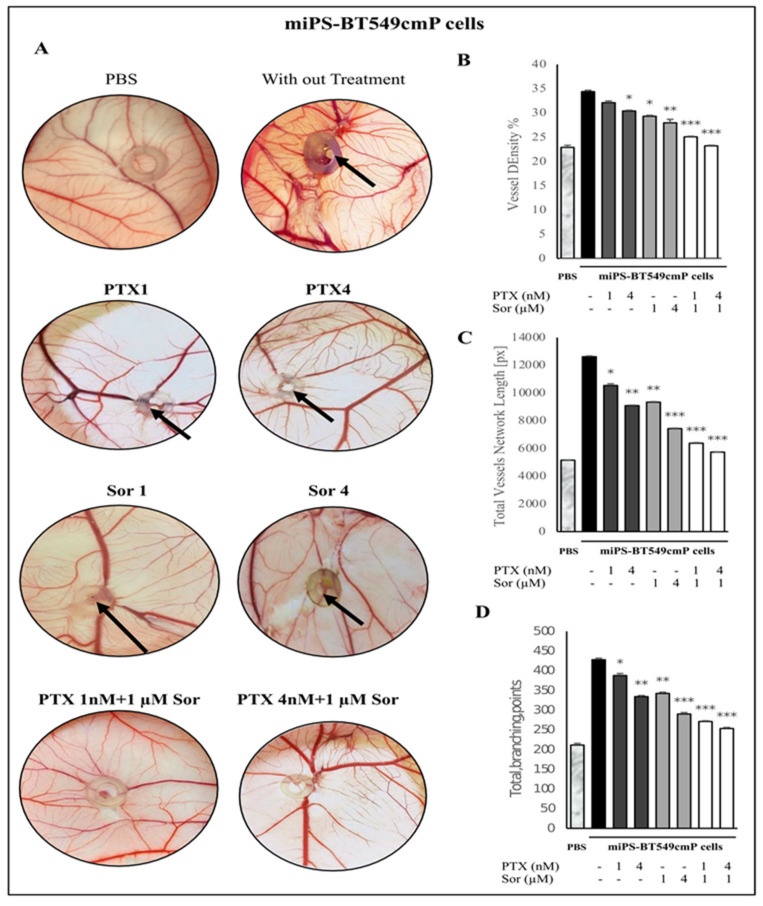
In vivo CAM assay. (**A**) Macroscopic observations of the antiangiogenic effect of combined treatment of PTX and Sor on miPS-BT549cmP cells. Numerous allantoic vessels converge radially around non-treated controls. Following combined treatment with PTX and Sor, evident reduction in neovessel formation compared with single treatment groups. In addition, inhibition of tumor formation was found in combined groups compared with single treatment groups (black arrow). (**B**–**D**) Analysis of photographs from (**A**) of the vessel density (**B**), total vessel length (**C**), and number of branching points of the vessels (**D**) of the CAM illustrating the anti-angiogenic effect of single or combined treatment of PTX and Sor of miPS-BT549cmP cells.

In order to investigate the antiangiogenic effect of the combined treatment of PTX and Sor on miPS-Huh7cmP and miPS-BT549cmP cells in vivo, the chick chorioallantoic membrane (CAM) assay was utilized in this study. Examination was performed at day 13 of incubation after 4 days of injection. The results demonstrated that the controls, either miPS-Huh7cmP (Figure 7A) or miPS-BT549cmP cells (Figure 8A), showed the most angiogenic potency with numerous allantoic vessels radiating around the injected cells with abnormal radiating morphology. However, miPS-Huh7cmP and miPS-BT549cmP cells subjected to combination treatment with PTX (either 1 or 4 nM) and Sor (1 µM) showed the most antiangiogenic activity inhibiting neo-vascularization. The combination drastically reduced the number of radiating secondary vessels compared with PTX single treated cells. Interestingly, the high dose of combined treatment (PTX 4 nM + Sor 1 µM) resulted in the most potent antiangiogenic effect with a vascularization pattern closer to the PBS injected group with complete inhibition of newly formed blood vessels. Meanwhile, CSCs, miPS-Huh7cmP, and miPS-BT549cmP cells, applied on CAM without drug treatments or with one drug treatment, exhibited tumor formation ability which was noticed as a tumor mass on CAM. However, combined treatment resulted in no tumor formation, suggesting the efficacy of combined PTX and Sor on inhibition of tumor initiation ability. These results indicate the potential antiangiogenic and tumor formation inhibition effects of combined treatment with PTX and Sor on miPS-Huh7cmP and miPS-BT549cmP cells.

The antiangiogenic effect of combined treatment of PTX and Sor on either miPS-Huh7cmP or miPS-BT549cmP cells in chick CAM was evaluated through image analysis using the WimCam software program (Wimasis, Munich, Germany). Inhibition of angiogenesis was evaluated based on three parameters, percentage of vessel density, total vessels network length, and sprouting ability indicated by the total branching points. Table S1 and Figure 7B–D show that combined treatment of PTX and Sor resulted in a high significant decrease of the vessel density (Figure 7B), total vessels network length (Figure 7C), and total branching points (Figure 7D) of the chick CAM inoculated with miPS-Huh7cmP cells compared with non-treated cells. Moreover, the high dose of combined treatment (PTX 4 nM + Sor 1 µM) showed the most antiangiogenic effect compared with all other groups. Similarly, Table S2 and Figure 8B–D show the evaluation of angiogenesis inhibition potency of either single or combined treatment of PTX and Sor on miPS-BT549cmP cells in the chick CAM model. The results showed a high significant decrease in the angiogenesis when the cells were treated with both PTX and Sor, in addition, high dose of combined treatment (PTX 4 nM + Sor 1 µM) resulted in the least angiogenic effect in terms of vessel density (Figure 8B), total vessels length (Figure 8C), and total branching points (Figure 8D) and showed highly significant results compared with non-treated control cells. All these data demonstrate that combined treatment of PTX and Sor, especially the high dose, significantly decreases the angiogenesis effect of miPS-BT549cmP and miPS-Huh7cmP cells in the in vivo chick CAM model.

## 4. Discussion

Although chemotherapy is one of the main treatments for cancer patients, chemo-resistance, which limits the efficiency of anti-cancer agents, is a major problem as cancer cells often become resistant to chemical substances used in treatment [18]. This is considered due to the heterogeneity resulting in cancer progression [5,19]. Accumulating evidence suggests that the CSC population, a subgroup of cancer cells, is responsible for the chemo-resistance and cancer relapse [20], as CSCs are plastic to differentiate into the heterogeneous lineages of cancer cells in response to the microenvironment including chemotherapeutic treatment. Many anti-cancer drugs are not free from side effects while they are approved for the treatment of cancer. Taking these into consideration, combination therapy sounds more feasible to expect more effective treatments for cancer targeting different pathways by which cancer cells survive and grow [21]. The ideal combination promotes the efficiency of monotherapies in lower doses of drug escaping from the toxicity and side effects. Studies have demonstrated the results of combining chemotherapeutic agents with different functions such as different tyrosine kinase inhibitors [22,23,24].

PTX, widely used as anti-cancer chemotherapy, is well known to stabilize microtubule binding to β-subunit of tubulin and to block the dynamics [25]. However, PTX is not efficiently available in high doses due to the insolubility to water and develops various side effects when used in treatments. In this context, reducing PTX doses would be more feasible to treat cancers and overcoming side effects of PTX.

Previously, our group demonstrated that CSCs can be developed from iPSCs in the presence of CM prepared from various cancer cell lines [10,11,12,14]. Since our goal in this study was to reveal the synergistic action of PTX and Sor at low doses for growth inhibition of CSCs, we used our model of CSCs developed from iPSCs [12,14] to assess the effect of the combination at low concentration. The effect of the combination was evaluated on self-renewal, clonogenic, differentiation potential of CSCs, as well as proliferation.

Sor in low doses enhanced the effect of PTX and decreased the IC_50_ values almost 10-fold, indicating that this drug combination should offer a new approach of targeting CSCs with fewer side-effects. Simultaneously, self-renewal potential was affected by PTX in the presence of a non-toxic level of Sor 1 µM. PTX and Sor suppressed the self-renewal of CSCs synergistically, while miPS-BT549cmP cells appeared slightly more resistant than miPS-Huh7cmP cells.

The combination exhibited a suppressive effect on the clonogenic and differentiation potential of CSCs at the same concentration and conditions used in self-renewal. In the case of colony formation, potential cotreatment synergistically inhibited colony formation compared with each agent alone. On the contrary sphere formation, miPS-Huh7cmP cells were more resistant than miPS-BT549cmP and tolerant to higher doses of both drugs.

The combination of PTX and Sor also inhibited angiogenesis with less network formation. Results showed that miPS-BT549cmP is resistant to either PTX or Sor but not resistant to the combination when compared with miPS-Huh7cmP. But a combination of PTX and Sor in general suppresses the ability of CSCs to form vessels. In vitro data that suggested the inhibition of self-renewal ability by combination treatment were confirmed by CAM assay, which showed the efficiency of PTX and Sor combination for inhibiting tumor formation ability of CSCs. Not only angiogenesis was inhibited, but also the ability of initiation tumor by the combination of PTX and Sor.

In this study, mitotic arrest, which should lead to apoptosis, was not observed. At low concentration, PTX causes G1-like arrest [26]. In this case, PTX appears to allow the first cell cycle not preventing cells from passing through S phase and entering mitosis. Although the M-pahse is prolonged, cells still divide, demonstrating the presence of a sub G1 cells caused by low concentration of PTX. It is important to note that the sub G1 cells were viable and non-apoptotic. Simultaneously, sorafenib inhibits tyrosine kinase activity leading to RAF/MEK/ERK signaling pathway stimulating cell growth [27,28]. This suggests that sorafenib suppresses the cells entering into S-phase. In the condition of our result, the concentration of sorafenib was so low that the cells should enter the first cell cycle, but the entrance might be delayed. Thus, post-mitotic arrest should be occurring in the second and even the third cell cycles as the result of the synergistic effect of PTX and sorafenib.

The combination of Sor and PTX was demonstrated to have a positive effect on anti-angiogenesis in vivo in metastatic breast cancer [29]. If this observation was related to the differentiation potential of CSCs, our results may agree with the previous report. Very recently, triple combination of radiation, Sor, and PTX has been reported to be effective on breast cancer cell lines [30]. Further investigation for the effects of radiation and the combination of Sor and PTX on CSCs will be interesting as CSCs are considered to be resistant to radiation therapy.

## 5. Conclusions

The combination of PTX and Sor showed a synergistic effect on our original CSC models in vitro. The combination was found significant to suppress the properties of CSCs. These results propose a novel approach to target CSCs with anticancer drugs in low doses, which would effectively reduce the toxic side effects of chemotherapy.

## Supplementary Materials

The following are available online at https://www.mdpi.com/2072-6694/12/6/1360/s1, Figure S1: Representative images showed the effect of combined treatment of PTX and Sor on Huh7cells, Table S1: Showing the antiangiogenic effect of single or combined treatment of Ptx and/or Sor on miPS-Huh7cmP cells in the chick CAM, Table S2: Showing the antiangiogenic effect of single or combined treatment of Ptx and/or Sor on miPS-BT549cmP cells in the chick CAM.

## Abbreviations

CSCs	cancer stem cells
iPSCs	induced pluripotent stem cells
CM	conditioned medium
PTX	paclitaxel
Sor	sorafenib
miPS	mouse induced pluripotent stem cells
PBS	phosphate buffer solution
miPS-Huh7cmP	primary cultured cells isolated from liver tumor
miPS-Bt549cmP	primary cultured cells derived from tumor under skin
GFP	green fluorescent protein
LIF	leukemia inhibitory factor
Puro	puromycin
FBS	fetal bovine serum
DMSO	dimethyl sulfoxide
NEAA	non-essential amino acids
PI	propidium iodide
STR	profiling: short tandem repeat
hEGF	human epidermal growth factor
R3-IGF	R3-insulin-like growth factor-1
hbFGF	human basic fibroblast growth factor
VEGF	vascular endothelial growth factor
GA	gentamicin/amphotericin B
CAM	chorioallantoic membrane assay
