# Supplementary material for: Paclitaxel and Sorafenib: The Effective Combination of Suppressing the Self-Renewal of Cancer Stem Cells
Source: Cancers (Basel). 2020 May 26;12(6):1360. doi: 10.3390/cancers12061360 (PMC7352607; doi:10.3390/cancers12061360)
Supplement: Supplementary file 1 [file cancers-12-01360-s001.pdf]

Supplementary Material

Paclitaxel and Sorafenib: the effective combination of suppressing the self-renewal of cancer stem cells

Hend M Nawara, Said M. Afify, Ghmkin Hassan, Maram H Zahra, Marwa N Atallah, Hager Mansour, Hagar A Abu Quora, Md Jahangir Alam, Amira Osman, Hiroki Kakuta, Hiroki Hamada, Akimasa Seno and Masaharu Seno

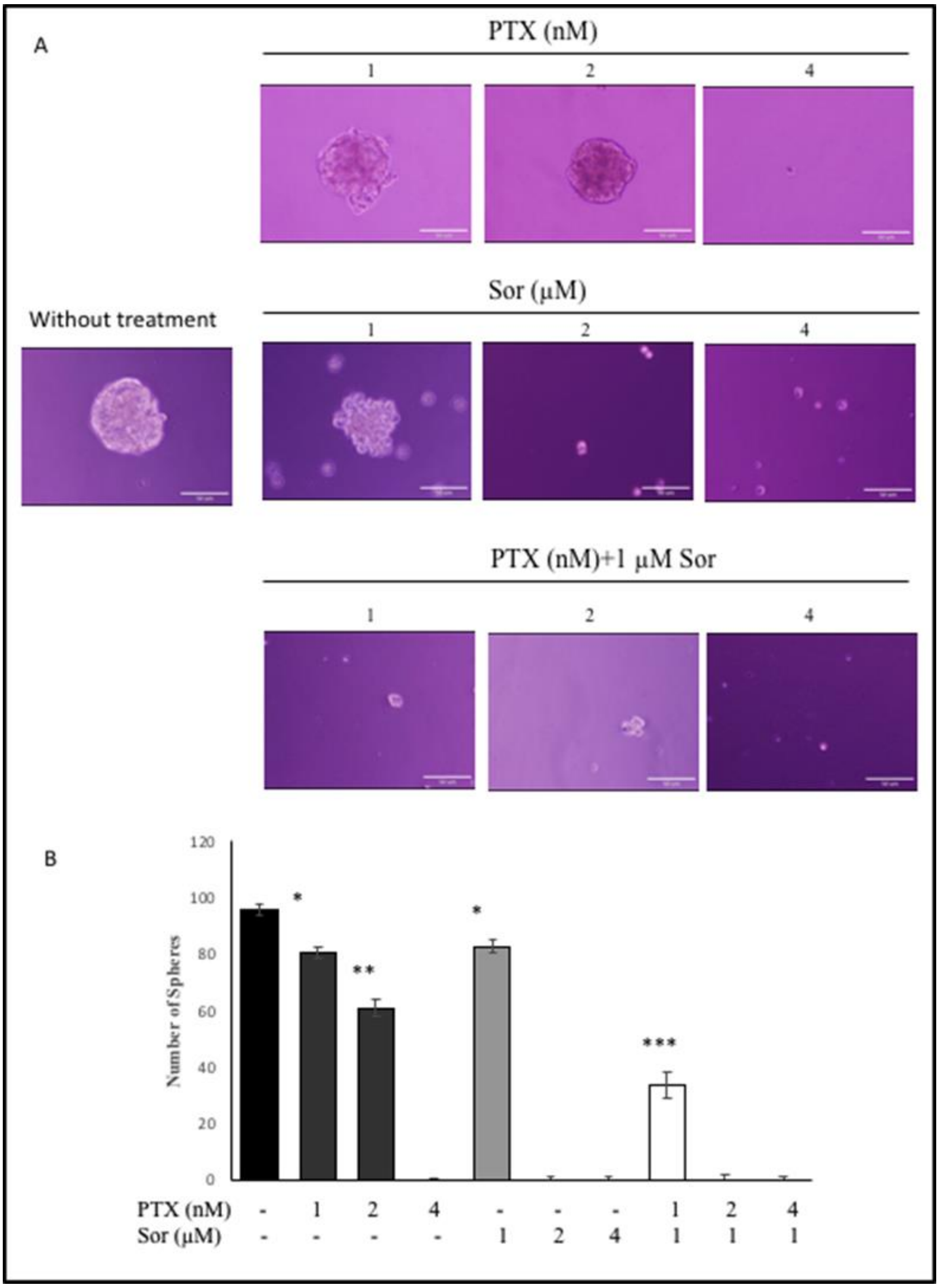

Figure S1. Representative images showed the effect of combined treatment of PTX and Sor on Huh7cells. The asterisks represent the mean expression levels; * $p < 0.05$; ** $p < 0.001$; *** $p < 0.0001$.

Table S1. Showing the antiangiogenic effect of single or combined treatment of Ptx and/or Sor on miPS-Huh7cmP cells in the chick CAM. The asterisks represent the mean expression levels; *$p < 0.05$; **$p < 0.001$; ***$p < 0.0001$.

Mean ± SD	Vessel Density (%)	Total Vessels Network Length [px]	Total Branching Points
PBS	22.9 ± 0.430	5152 ± 21.40	211 ± 4.27
HUH Control	31.4 ± 0.270	12097 ± 39.32	397 ± 3.89
HUH-P1	29.7 ± 0.083	9861.8 ± 26.66*	321 ± 4.50*
HUH-P4	28.6 ± 0.268*	8532 ± 67.33**	295 ± 7.36*
HUH-S1	28 ± 0.192*	8913.4 ± 14.48*	305 ± 3.11*
HUH-S4	27.1 ± 0.158**	7329.8 ± 36.34**	274 ± 5.54**
HUH-C1	24.6 ± 0.268***	6028.2 ± 21.89***	248 ± 8.01***
HUH-C4	22.9 ± 0.151***	5454.4 ± 38.81***	227 ± 4.15***

Table S2. Showing the antiangiogenic effect of single or combined treatment of Ptx and/or Sor on miPS-BT549cmP cells in the chick CAM. The asterisks represent the mean expression levels; * $p < 0.05$; ** $p < 0.001$; *** $p < 0.0001$.

Mean ± SD	Vessel Density (%)	Total Vessels Network Length [px]	Total Branching Points
PBS	22.9 ± 0.430	5152 ± 21.40	211 ±4.27
BT Control	34.4 ± 0.273	12621.4 ± 55.40	428 ± 3.80
BT-P1	32.1 ± 0.349	10543.2 ± 125.49*	387 ± 5.16*
BT-P4	30.4 ± 0.158*	9092.4 ± 37.10**	334 ± 2.79**
BT-S1	29.3 ± 0.192*	9339.8 ± 37.85**	342 ± 3.20**
BT-S4	27.9 ± 0.723**	7432.6 ± 36.37***	290 ± 3.76***
BT-C1	25.0 ± 0.130***	6378 ± 45.51***	271 ± 1.92***
BT-C4	23.2 ± 0.130***	5736.2 ± 18.95***	253 ± 2.79***

© 2020 by the authors. Licensee MDPI, Basel, Switzerland. This article is an open access article distributed under the terms and conditions of the Creative Commons Attribution (CC BY) license (http://creativecommons.org/licenses/by/4.0/).
